# Risk factors for esophagitis after hypofractionated palliative (chemo) radiotherapy for non-small cell lung cancer

**DOI:** 10.1186/s13014-020-01550-2

**Published:** 2020-05-01

**Authors:** Carsten Nieder, Kristian S. Imingen, Bård Mannsåker, Rosalba Yobuta, Ellinor Haukland

**Affiliations:** 1 Department of Oncology and Palliative Medicine, Nordland Hospital, 8092 Bodø, Norway; 2 Department of Clinical Medicine, Faculty of Health Sciences, University of Tromsø, 9037 Tromsø, Norway

**Keywords:** Lung cancer, Radiotherapy, Chemoradiation, Palliative radiation therapy, Esophagitis, Dose-volume histogram

## Abstract

**Introduction:**

Esophagitis influences quality of life and might cause treatment interruption and hospitalization. Previous studies of risk factors focused on curative treatment for non-small cell lung cancer (NSCLC), which often involves concomitant chemoradiation (CRT). Given the uncertainty around extrapolation of dose constraints, we analyzed risk factors in patients treated with hypofractionated palliative regimens.

**Patients and methods:**

A retrospective review of 106 patients treated with palliative radiotherapy or CRT between 2009 and 2017 was performed. Inclusion criteria: prescribed total dose 30–54 Gy, dose per fraction 2.5–4 Gy, esophageal dose > 1 Gy. Uni- and multivariate analyses were performed in 97 eligible patients to identify predictive factors for acute esophagitis grade ≥ 1 (CTCAE 5.0).

**Results:**

Forty percent of patients were treated with 15 fractions of 2.8 Gy (42 Gy) and 28% also received chemotherapy according to the CONRAD study regimen (induction and concomitant Carboplatin/Vinorelbine) published by the Norwegian Lung Cancer Group. Thirty-four percent were treated with 10 fractions of 3 Gy. Stage IV NSCLC was present in 47%. Esophagus Dmax was 39 Gy (population median) and Dmean 15 Gy. Overall 31% of patients developed esophagitis (26% grade 2–3, no grade 4–5). Several dosimetric parameters correlated with the risk of esophagitis (Dmax, Dmean, D5cc, V20, V30, V35, V40). Dmax outperformed other dosimetric variables in multivariate analysis. Furthermore, concomitant chemotherapy significantly increased the risk of esophagitis, while oral steroid medication reduced it. In patients with Dmax ≥40 Gy a reduced Dmean (≤20 Gy) was beneficial.

**Conclusion:**

In order to reduce esophagitis after hypofractionated palliative treatment lower doses than those recommended in curative NSCLC settings are preferable. Besides esophageal dose, CRT is the main risk factor for esophagitis. Additional work is needed to confirm that steroids are able to modify the risk (or to rule out confounding effects of baseline variables not included in our database).

## Introduction

Multimodality treatment of non-small cell lung cancer (NSCLC) has become more effective in recent years, e.g., due to stereotactic ablative radiotherapy for stage I disease [1, 2] and oligometastatic stage IV disease [3–5], and immunotherapy for stage III and IV disease [6, 7]. Patients with stage III disease who are unfit for radical chemoradiotherapy should be considered for reduced-intensity palliative chemoradiotherapy [8–10]. One of the downsides of chemoradiotherapy is its potential to induce painful acute esophagitis [11–13]. In the context of radical or curative chemoradiotherapy, typically for stage III NSCLC, several studies have addressed the incidence, time course and impact on quality-of-life of radiation-induced esophagitis [14]. In addition, risk factors, predictive models and dose constraints have been proposed [15, 16]. However, limited efforts have been made to characterize esophageal toxicity and dosimetric risk factors during and after hypofractionated palliative (chemo) radiotherapy [17]. The aim of the present single-institution study was to provide additional clinical data about this topic.

## Patients and methods

A retrospective review of 106 patients treated with palliative 3-dimensional conformal radiotherapy or chemoradiotherapy between 2009 and 2017 was performed. Inclusion criteria: prescribed total dose 30–54 Gy, dose per fraction 2.5–4 Gy (once daily, 5 fractions per week), maximum esophageal dose (Dmax, maximum point dose) > 1 Gy. Based on these, 97 patients were eligible for further evaluation and 9 were excluded (because of low esophageal radiation dose). In case of chemoradiotherapy, most patients received the Norwegian CONRAD regime (15 fractions of 2.8 Gy, Carboplatin/Vinorelbine before and during radiotherapy) [18]. Uni- and multivariate analyses were performed to identify predictive factors for acute esophagitis grade ≥ 1 (CTCAE 5.0; physician assessed), e.g. mean (Dmean) and maximum dose, dose to 5 and 10 cc, volume exposed to 20 (V20) and 30 Gy etc. Clinical information throughout follow-up after treatment was abstracted from our electronic patient record system in order to capture both acute (first 3 months) and late toxicity (after more than 3 months), e.g. stenosis or perforation not caused by the cancer itself. All patients were followed during treatment by oncology nurses and physicians, including assessment of esophagitis at the end of radiotherapy and 6–8 weeks later. Weight loss was not recorded in detail. Symptoms from acute esophagitis were palliated as needed, e.g. with analgetics. After the first follow-up visit, intervals were increased to 3 months. Treatment plans were calculated with Varian Eclipse TPS® and no intensity-modulated or arc-based techniques (IMRT, VMAT) were employed. Respiratory motion management or adaptive treatment was not employed either. Mandatory organs at risk included spinal cord and lungs, whereas contouring of the esophagus was left to the discretion of the oncologist. If contoured, no standardized dose constraint or field set-up was employed. For patients with unavailable dosimetric data (46%), the esophagus (including contents and/or air) was contoured retrospectively on the treatment planning scans for the purpose of this study (from the upper border of the lung to the diaphragm, identical to already contoured cases). IBM SPSS v.25 was employed for statistical analyses. The latter included chi-square test and binary logistic regression for associations between esophagitis (yes/no) and clinical and dosimetric variables. Significant variables, i.e. *p* < 0.05, were then included in multi-nominal logistic regression analysis.

## Results

The median age was 70 years, range 41–90. Fifty-nine patients (61%) were men. Stage and histology distribution were as follows: less than III in 4%, III in 49%, IV in 47%, adenocarcinoma in 44% and squamous cell carcinoma in 41% (other or unspecified in 15%). T3 and T4 tumors were treated in 35 and 32%, respectively (N2: 50%, N3: 28%). Only 5% were never smokers and 12% active smokers at the time of radiotherapy. Forty percent of patients were treated with 15 fractions of 2.8 Gy (42 Gy) and 28% also received chemotherapy according to the CONRAD study regimen. Thirty-four percent were treated with 10 fractions of 3 Gy. Five percent each received 12 and 15 fractions of 3 Gy, respectively. The remaining patients were treated with different other fractionations. Forty-five percent were chemotherapy-naϊve when they received radiotherapy. Any type of oral steroid medication was used concomitant to radiotherapy in 42% of patients. Reasons included comorbidity, reduced appetite, presence of brain metastases etc. Dose and duration of steroid treatment varied.

Overall 31% of patients developed acute esophagitis (26% grade 2–3, no grade 4–5). Esophagitis did not cause any treatment interruption or termination. However, 2 patients (2%) were hospitalized because of esophagitis shortly after completion of radiotherapy. Three patients (3%) did not receive further chemotherapy because of esophagitis with weight loss and reduced general condition. Serious complications such as esophageal perforation or fistula were not observed. Later during follow-up 3% developed esophageal obstruction, always as result of tumor progression.

As shown in Table 1, esophagus Dmax was 39.1 Gy (population median) and Dmean 15.3 Gy. As displayed in Table 2, several dosimetric parameters correlated with the risk of esophagitis (Dmax, Dmean, D5cc, V20, V30, V35, V40). Furthermore, concomitant chemotherapy significantly increased the risk of esophagitis (e.g., for Dmax > 39 Gy from 23 to 46%), while oral steroid medication reduced it. Age, gender and smoking were not associated with esophagitis. The significant variables shown in Table 2 were carried forward to multi-nominal logistic regression analysis, which demonstrated that concomitant chemotherapy (odds ratio 3.7 (1.1–12.4), *p* = 0.03), lack of steroid medication (odds ratio 4.4 (1.1–17.2), *p* = 0.04) and Dmax (odds ratio 1.1 (1.01–1.20), *p* = 0.025) were associated with higher rates of esophagitis.

**Table 1:** Dosimetric parameters (doses in Gy, based on dose-volume histograms)

Parameter	Median	Range
CTV in cc	142	10–1185
PTV in cc	427	95–1950
Esophagus volume in cc	25	11–62
Esophagus volume inside PTV in cc	8.7	0–22
Maximum dose to esophagus	39.1	1.8–52.1
Mean dose to esophagus	15.3	0.7–37.0
Dose to 5 cc of esophagus	30.0	1.2–51.0
Dose to 10 cc of esophagus	22.0	0.0–51.0
Esophagus volume exposed to 20 Gy (V20)	40	0–79
Esophagus volume exposed to 30 Gy (V30)	24	0–73
Esophagus volume exposed to 35 Gy (V35)	15	0–72
Esophagus volume exposed to 40 Gy (V40)	0	0–60
Esophagus volume exposed to 50 Gy (V50)	0	0–16

*CTV* Clinical target volume, *PTV* Planning target volume

**Table 2:** Risk factors for esophagitis (yes/no; grade 1–3 combined), univariate analysis

Parameter	Odds ratio (95% confidence interval)	*p*-value
Concomitant chemotherapy (yes/no)	5.99 (2.30–15.50)	0.0001*
Concomitant steroid use (no/yes)	3.00 (1.13–7.94)	0.023*
Dmax esophagus	1.13 (1.04–1.23)	0.004**
Dmean esophagus	1.10 (1.03–1.18)	0.002**
V20 esophagus	1.04 (1.01–1.07)	0.001**
V30 esophagus	1.04 (1.01–1.06)	0.001**
V35 esophagus	1.04 (1.01–1.06)	0.001**
V40 esophagus	1.04 (1.01–1.07)	0.002**
Dose to 5 cc of esophagus	1.07 (1.02–1.12)	0.005**

Not significant: age, sex, T stage, N stage, smoking, history of gastroesophageal reflux disease, dose to 10 cc esophagus, esophagus volume inside PTV

* Chi-square test

** Binary logistic regression analysis

**Fig. 1:**
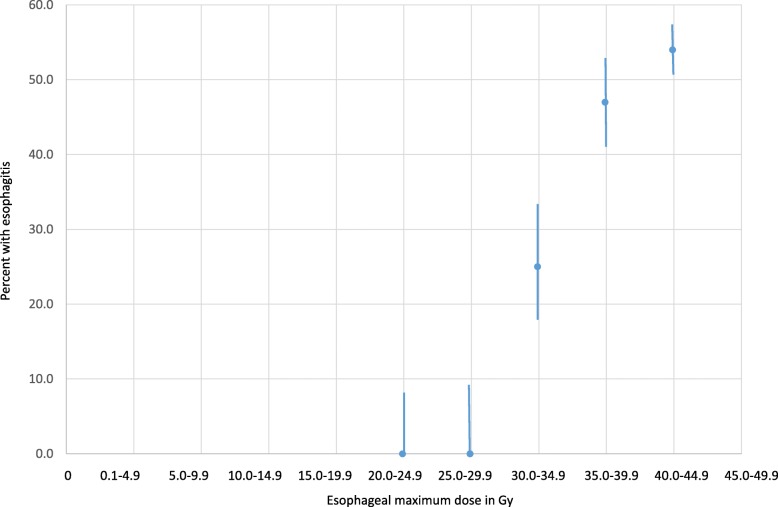
Risk of esophagitis (yes/no; grade 1–3 combined) after different maximum doses to the esophagus (< 25 Gy, 25–29.9 Gy, 30–34.9 Gy, 35–39.9 Gy, 40 Gy or more)

As illustrated in Fig. 1, the risk of esophagitis increased if the maximum dose to the esophagus exceeded 30 Gy, and in particular if it approached 40 Gy. In patients with Dmax ≥40 Gy a reduced Dmean was beneficial. With Dmean ≤20 Gy 65% of patients remained free from esophagitis, compared to only 31% if Dmean exceeded 20 Gy (*p* = 0.02, 2-tailed Fisher exact probability test). Median actuarial overall survival (Kaplan-Meier method) was 12 months in the chemoradiotherapy cohort and 7 months after radiation alone (log-rank test *p* = 0.05).

## Discussion

Palliative (chemo) radiotherapy is an important component of care for many patients with NSCLC [8]. Sequential plus concurrent palliative chemoradiotherapy improves survival compared with chemotherapy alone [18], but it increases toxicity, particularly radiation esophagitis. More than 85% of the patients receiving chemoradiotherapy in the CONRAD study reported various degrees of esophagitis, but none reported grade 4 [18]. Validated predictors of esophagitis for clinical use in this population are lacking. In the curative setting, an individual-patient-data meta-analysis has been performed [19]. Factors predictive of esophagitis grade ≥ 2 and grade ≥ 3 were assessed. Most patients received platinum-containing regimens. The development of esophagitis was common, scored as grade 2 in 32%, grade 3 in 17%, and grade 4 in 1%. On univariable analysis several baseline factors were statistically predictive of esophagitis, but only dosimetric factors had good discrimination scores. On multivariable analysis, the esophageal volume receiving ≥60 Gy (V60) alone emerged as the best predictor of grade ≥ 2 and grade ≥ 3 esophagitis. Additional research is needed for palliative scenarios, which typically employ hypofractionated regimens with moderate total doses (often 30–45 Gy). Despite dose reduction, esophagitis influences quality of life and might cause weight loss, treatment interruption and, in severe cases, hospitalization [18].

The RTOG 0617 study compared curative standard-dose (60 Gy) versus high-dose (74 Gy) radiation with concurrent chemotherapy and determined the efficacy of cetuximab for stage III (NSCLC) [20]. The study used a 2 × 2 factorial design with radiation dose as one factor and cetuximab as the other. Treatment-related grade ≥ 3 dysphagia and esophagitis occurred in 3 and 5% of patients in the 60-Gy-arm versus 12 and 17% in the 74-Gy-arm, respectively (*p* = 0.0005 and < 0.0001). Factors associated with improved overall survival on multivariable analysis were standard radiation dose, tumor location, institution accrual volume, esophagitis/dysphagia, PTV and heart V5. Thus, this study supports treatment planning efforts leading to lower esophageal and heart doses.

The aim of our study was to identify predictive factors for acute esophagitis in the non-curative setting. We reviewed the treatment planning and clinical data, and decided to dichotomize esophagitis (yes/no), because its overall incidence was limited and few patients developed grade 3 toxicity, meaning that the statistical power of separate analyses for grade 2 or 3 would have been very limited. The grading was based on retrospective chart review, a limitation which is shared with many previous studies, which might lead to underestimated rates of mild toxicity. Prospective assessment, as done in the CONRAD study, would have provided more detailed information about the patients’ nutrition status and symptom burden. Because almost all treatments employed 2.8–3.0 per fraction, we did not convert doses to biologically equivalent doses, such as EQD2.

In line with previous research, we found associations between several dosimetric factors and risk of esophagitis. The most important of these risk factors was maximum dose to the esophagus. As illustrated in Fig. 1, doses lower than 35 Gy are recommended (as low as reasonably achievable without exceeding lung and spinal cord tolerance). However, the presence of N2 and N3 lymph node metastases might complicate sparing of the esophagus, due to anatomical proximity, even if 3-D conformal techniques are replaced by intensity-modulated and/or arc-based approaches [21–24]. In own patients with high Dmax, a reduction of Dmean was associated with lower rates of esophagitis. It is known from previous studies that concomitant chemoradiotherapy increases esophageal toxicity, as also seen in our analysis. Unexpectedly, concomitant oral steroids reduced the risk of esophagitis. Unless confirmed in a prospective trial, one has to consider the influence of confounding factors and possible interactions between steroid use and lack of chemotherapy or prescription of lower doses of radiotherapy. Given that many of our patients had stage IV NSCLC it appears possible that other concomitant medications were used, e.g. analgetics, which were not captured in this study. Baseline use of analgetics would probably mask the symptoms from mild esophagitis, especially if not evaluated with prospectively administered patient questionnaires.

A recent single arm trial (ICORG 06–34) studied 3-D conformal radiotherapy to reduce the toxicity of palliative lung irradiation [17]. Fractionation regimens included 39 Gy in 13 fractions, 20 Gy in 5 fractions, and 17 Gy in 2 fractions. The primary endpoint was the occurrence of grade 3 or higher esophagitis (CTCAE v4.02). The mean dose to the irradiated esophagus, defined as one centimeter above and below the PTV, should be ≤30% of the reference point dose, unless the PTV was extending into the esophagus. Overall, 30 patients could be analyzed (22 treated with 20 Gy, 12 with 39 Gy, 1 with 17 Gy). For the 39 Gy regimen Dmax was 23–42 Gy (mean 38 Gy), while the mean dose to the irradiated esophagus was as intended (mean 23 Gy). No grade 3 esophagitis was recorded. Grade 2 toxicity did not exceed 11% either. Due to contouring difference (irradiated vs. whole esophagus) and study design (prospective vs. retrospective) these data are difficult to compare to ours. Nevertheless, both studies suggest that efforts to spare the esophagus should be pursued. In the future, larger databases should be established in order to develop more advanced prediction models. As a consequence of Fig. 1, our current treatment planning strategy is to limit Dmax to the esophagus, e.g. by accepting a PTV coverage of < 95% at the intersection with the esophagus. If a high Dmax is unavoidable, we try to reduce Dmean.

## Conclusion

In order to reduce acute esophagitis during and after hypofractionated palliative treatment, lower doses than those recommended in curative NSCLC settings are preferable. Besides esophageal dose, CRT is the main risk factor for esophagitis. Additional work is needed to confirm that steroids are able to modify the risk (or to rule out confounding effects of baseline variables that were not included in this study).

## Data Availability

The dataset supporting the conclusions of this article is available at request from the corresponding author, if intended to be used for meta-analyses.

## Abbreviations

CRT: Chemoradiotherapy
Dmax: Maximum dose
Dmean: Mean dose
NSCLC: Non-small cell lung cancer
